# Supplementary material for: Are carbon emissions trading and green financial instruments synergistic? -Comprehensive quantitative research based on content analysis
Source: PLoS One. 2024 Mar 7;19(3):e0298601. doi: 10.1371/journal.pone.0298601 (PMC10919601; doi:10.1371/journal.pone.0298601)
Supplement: S1 Data — (ZIP) [file pone.0298601.s001.zip › Data/地级市Z_F工作报告.zip!/┴╔─■/│»╤⌠╩╨/└┤╫╘╣1⁄2╓┌║┼í╛┬φ┐╦╩2╛▌═°í┐_2019.docx]

各位代表：

现在，我代表市人民政府向大会报告工作，请予审议，并请各位政协委员和其他列席人员提出意见。

2018年工作回顾

2018年是本届政府的开局之年。我们以习近平新时代中国特色社会主义思想为指导，全面贯彻党的十九大和十九届二中、三中全会精神，深入落实习近平总书记在辽宁考察时和在深入推进东北振兴座谈会上的重要讲话精神，在省委、省政府的亲切关怀和市委的正确领导下，在市人大及其常委会和市政协的监督支持下，团结带领全市各族人民，坚持稳中求进工作总基调，按照高质量发展要求，持之以恒践行新发展理念和“四个着力”“三个推进”，三大攻坚战开局良好，供给侧结构性改革扎实推进，人民生活持续改善，保持了经济持续健康发展和社会大局稳定，朝着实现全面建成小康社会的目标迈出新的步伐。

一、经济健康稳定增长

紧紧扭住第一要务，全力推进经济发展，统筹做好稳增长、促改革、调结构、惠民生、防风险工作，全市经济运行呈现稳中有进、总体向好的态势。预计完成地区生产总值834亿元，增长5%；一般公共预算收入70.4亿元，增长21.7%，增速居全省首位；固定资产投资256亿元，增长2%；社会消费品零售总额477.6亿元，增长5.5%；城乡常住居民人均可支配收入分别达到25600元和12840元，分别增长7%和8%。

用市场化手段推进“五个一”开放合作平台发展，推行“管委会、公司”模式，创新用人、薪酬、考核机制，进一步强化基础设施建设，园区承载力、吸引力持续提升。建成标准化厂房307万平方米，入驻企业252家。

实行市县领导包扶重点项目责任制，建立“项目管家”制度，全力做好服务和要素保障工作。落实减税降费政策，为企业和群众减轻负担1000万元；284户企业参与直购电交易，节约用电成本2600万元。全年实施亿元以上建设项目197个、10亿元以上建设项目14个。

把扩大增量、盘活存量作为稳增长的重要手段，开展大型招商活动15次，引进内资150亿元、外资5900万美元。北票汽摩配产业园、凌源炜盛玻纤新材料、朝阳县阳光光缆光纤、建平洪海岩棉保温材料、喀左印刷包装产业园、双塔农产品加工仓储、龙城九凤玄武岩纤维等项目落地建设。制定支持政策，推动老企业转型升级和民营经济发展，一批老企业通过对外合资合作焕发生机和活力。

抢抓新一轮东北振兴等重大机遇，积极做好政策和项目争取工作，城区老工业区搬迁改造重点工程、采煤沉陷区综合治理试点以及军民融合试点示范项目获得国家支持。全年向上争取专项资金56亿元，同比增长9.5%。

二、结构调整深入推进

以实施乡村振兴战略为牵动，大力发展现代农业。调减玉米种植面积6.45万亩，新增设施农业7650亩。粮食喜获丰收，产量达26亿公斤。狠抓“一区一园一基地”发展，完成20个现代农业产业园和100个基地规划编制并全面启动建设，龙城农产品食品加工园区晋升国家农业科技园区。推进农产品深加工，市级以上龙头企业达到287家。进一步推动农业共营制建设，成立土地股份合作社1079家。

加快工业结构调整。一手抓老企业转型升级，一手抓战略新兴产业培育，新增高新技术企业20家、省级产业技术创新平台11家，高新技术产业增加值增长27%。关闭30万吨以下煤矿1处，化解产能21万吨。实施质量强市战略，8个产品获评省名牌。新增“小升规”企业3 9家。规模以上工业实现增加值135亿元，增长8.4%。

进一步壮大现代服务业。共有7个电商园投入使用，累计引进和培育电商企业612家、电商网点866个。肉菜流通追溯体系34个监测节点正式运营，13个特色产品获“辽宁礼物”、2个产品获“辽宁老字号”称号。完成商品房销售130万平方米。积极发展旅游业，大黑山、热水汤、龙源旅游区等景区对外合资合作深入推进，乡村旅游全面启动，旅游区域市场初步形成，旅游业总收入增长17.7%。发挥金融服务实体经济作用，实现社会融资82亿元。

三、改革创新实现突破

大力推进审批服务便民化和“一网一门一次”改革，政务服务事项网上可办率达到81.6%，“一门”集中办理率达到85.7%，70个高频事项实现“最多跑一次”，一般性经营企业开办时间压缩到3个工作日内。积极开展以“多证合一、一照通”为主的商事制度改革，进一步放宽市场准入，新增市场主体3.3万户。针对痛点堵点问题，集中开展“20、4”办事难专项整治，企业和群众满意度不断提升。

积极推进园区改革，进一步整合园区机构，优化园区功能，全市生产力布局向园区倾斜、产业向园区集中、要素向园区流动、企业向园区聚集的发展格局已经形成。

制定落实支持政策措施，“飞地经济”发展、县乡财政“分灶吃饭”等工作走在全省前列。乡镇场街落地“飞地”项目157个，全省“飞地经济”工作现场会在我市召开。实行新的县乡财政管理体制，139个乡级金库全部上线运行。全市乡镇一般公共预算收入同比增长37.4%。

加大国企改革力度，凌钢集团纳入国家国有企业改革“双百行动”，朝阳银行初步完成股权多元化改革、迈入全国城商行第二阵营。城发、水务、文旅集团基本实现稳定运营，金控、环境、交投、农垦集团挂牌成立。“三供一业”分离移交、厂办大集体改革顺利推进。纳入省考核口径的市属企业资产总额、营业收入和利润分别增长8.5%、17.6%和36.1%。

实施“大学进园区进企业”工程，177家企业和100所科研院所开展合作，落实技术合作项目209项。开展“双创”活动，新建“双创”平台7家。喀左县成为全国首批创新型县。制定16条人才新政，推进十大引才工程，引进专家团队101个、专业技术人员575人。

改革机构设置，优化职能配置，积极稳妥推进事业单位和政府机构改革，进一步提升了政府效能。

四、城乡面貌明显改善

以创建国家森林城市为抓手，完成造林绿化103万亩、水土流失综合治理101万亩。推进集中供热、拆除燃煤小锅炉、淘汰老旧车辆、秸秆禁烧等工作，空气优良天数达到285天。全面推行河长制，启动一河一策治理管护，新增污水处理能力3万吨。实施土壤污染防治行动计划，土壤环境质量进一步改善。坚决落实中央环保督察整改要求，306件信访投诉案件全部销号，“回头看”交办案件办结率97.5%，解决了一批群众关注的热点难点问题。

加强宜居乡村建设，农村生活垃圾处置体系覆盖40%行政村，现有畜禽规模养殖场粪污处理设施配套率达到78%。创建省级生态乡镇1个、生态村10个，完成“四好农村路”新建改建工程1700公里。

全力抓好外部侵害整治、交通枢纽建设及相关拆迁工作，京沈客专朝阳段正式通车运营，朝凌、喀赤客专建设进展顺利，锦承铁路、叶赤铁路扩能改造扎实推进。中心城区龙泉大街、新华南路等道路及管网改造升级项目按时完成，凌源市启动建设环城公路，建平县“一环四横八纵”城市路网体系基本形成。特别让我们振奋的是，随着京沈客专沈承段正式运营，我市跨入高铁时代，交通和区位条件发生革命性变化，必将迎来重大发展机遇！

五、风险隐患有效化解

通过组建企业集团、合理安排财政预算资金等方式，完成40亿元年度政府债务化解任务。采取增加财政投入、扩面征缴、加强管理等措施，千方百计筹措资金，为20.1万名企业退休职工按时足额发放养老金58亿元。推进地方法人银行机构不良资产风险化解、重点债务企业风险化解、公职人员拖欠银行贷款清理、非法集资风险防控、规范民间融资等工作，金融风险得到有效控制。

坚持以解决问题为核心，深入推进信访矛盾减存控增三年攻坚计划，筹措资金近10亿元，有效化解一批历史遗留重大信访案件、兑现一批政府承诺事项。强化安全生产、食品药品安全、交通安全等风险管控，进一步做好国家安全、应急管理工作。加强社会治安综合治理和防控体系建设，深入开展扫黑除恶专项斗争，依法打掉涉黑涉恶团伙18个。采取有效防控措施，非洲猪瘟疫情零发生。成立社会治理应急联动中心，整合37个职能部门进驻。开通8890政务便民服务平台，政务公开、市民诉求办理工作取得新成绩。坚决防范和惩治统计造假、弄虚作假，为推动高质量发展提供统计保障。

六、民生保障持续增强

坚持尽力而为、量力而行，财政支出用于民生比重达到75%。完善大扶贫格局，用足用好深度贫困县有关政策措施，实施“1、10”扶贫举措，脱贫攻坚取得决定性进展，完成年度4个贫困县摘帽、250个贫困村销号、4.6万贫困人口脱贫任务，贫困发生率由2017年的2.5%下降到1.7%。新增城镇就业1.85万人，城镇登记失业率控制在3.99%。城乡低保标准分别提高10%和12%，企业、机关事业单位退休人员养老金和城乡居民养老保险基础养老金分别提高5.9%、4.5%和27%，居民医疗保险补助标准和失业保险金标准分别提高8%和3%。实施农村危房改造8072户，完成棚户区改造6385套。新建改扩建城乡社区办公用房项目23个，改建公办敬老院14所。

新建改扩建公办幼儿园16所，义务教育学校大班额削减41%；改造农村义务教育薄弱学校98所，7个县（市）区全部通过义务教育发展基本均衡县国家认定；高中教育优质发展、职业教育和高等教育转型发展取得新进展。公立医院改革扎实推进，组建医联体18个，县域内就诊率达96.9%；所有乡镇实现公立卫生院全覆盖，基层公共卫生服务能力进一步增强。实施文化体育惠民工程，公共图书馆、博物馆、群众艺术馆、文化站、农家书屋、社区书屋免费开放，送戏下乡300场；建设体育健身点2900个、农民健身广场100处，我市健儿在十三届省运会上获得好成绩。

一年来，我们落实全面从严治党责任，扎实推进“两学一做”学习教育常态化制度化。坚持依法行政，严格执行市人大及其常委会决议决定，主动接受人大、政协监督，办理人大代表建议137件、政协提案219件，满意率均达100%。推进依法决策、民主决策、科学决策，深化行政执法体制改革，确保权力在阳光下运行。深入实施“重实干、强执行、抓落实”专项行动和“转作风、树形象”专项治理，不断强化执行力，攻下了一批项目、解决了一批难题、办成了一批实事。民族宗教、外事、侨务、人防、气象、地震、档案、双拥、援疆、老年人、妇女儿童和残疾人保障等工作也取得新进展。

各位代表，过去一年成绩的取得，是习近平新时代中国特色社会主义思想指引的结果，是省委、省政府关心支持的结果，是市委坚强领导的结果，是市人大、市政协及社会各界监督支持的结果，是全市上下共同努力的结果。在此，我代表市政府，向全市各族人民，向人大代表、政协委员、各民主党派、工商联、无党派及社会各界人士，向驻朝中省直单位、人民解放军和武警官兵，表示崇高的敬意和衷心的感谢！

各位代表，在总结成绩的同时，我们也清醒地看到我市发展还存在一些短板和问题。主要表现在：一是经济增长不快，部分指标增速低于全省平均水平、没有达到“十三五”规划进度要求，经济下行压力仍然较大。二是体制机制不优，国有企业活力不足，民营经济发展缓慢，重点领域和关键环节改革不到位，营商环境需要进一步改善。三是对外开放步伐不大，新上大项目和好项目少，有效投资不足，发展后劲不强。四是经济结构不合理，传统产业转型升级步伐缓慢，新动能支撑能力远未形成，发展质量有待进一步提升。五是群众在公共服务、生态环保、安全稳定等方面还有许多不满意的地方。城市管理不到位，物业不规范、道路破损、私搭乱建、交通拥堵、停车难、占道经营等问题还比较突出。六是面对新形势、新任务，有的干部思想不够解放，新发展理念树得不牢，风险防控能力不强，抓落实力度不大，存在不敢担当、不愿作为的现象。我们将高度重视这些问题，采取有力措施加以解决。

2019年工作安排

今年是新中国成立70周年，是全面建成小康社会、推进朝阳振兴发展的关键之年。当前经济运行稳中有变、变中有忧，外部环境复杂严峻，不稳定不确定因素依然较多。尽管经济面临下行压力，但我国经济发展健康稳定的基本面没有改变，支撑高质量发展的生产要素条件没有改变，长期稳定向好的总体势头没有改变。特别要看到，经过不懈努力，我市产业基础逐渐丰厚，软硬环境不断改善，发展动力持续增强，政治生态进一步向好，随着进入高铁时代，将迎来新的重要战略机遇期。只要扬长避短、发挥优势，就一定能走出一条高质量发展的振兴之路！

今年政府工作的总体要求是：以习近平新时代中国特色社会主义思想为指导，全面贯彻党的十九大和十九届二中、三中全会精神，认真落实习近平总书记在辽宁考察时和在深入推进东北振兴座谈会上的重要讲话精神，统筹推进“五位一体”总体布局，协调推进“四个全面”战略布局，按照中央、省委经济工作会议和市委十届九次全会暨经济工作会议的安排部署，坚持稳中求进工作总基调，坚持新发展理念，坚持推动高质量发展，坚持以供给侧结构性改革为主线，坚持深化改革扩大开放，全力实施“161”重点工程，继续打好三大攻坚战，进一步做好稳增长、促改革、调结构、惠民生、防风险工作，促进朝阳全面振兴和全方位振兴。

主要预期目标是：地区生产总值增长6.5%，一般公共预算收入增长7%，固定资产投资增长8%，社会消费品零售总额增长7%，城乡常住居民人均可支配收入分别增长6.5%和7%。城镇登记失业率控制在4.5%以内。

一、以项目为中心和重心，不断培育新增长点

把“五个一”开放合作平台建成改革开放高地。以7个省级经济开发区为重点，深入推进“管委会、公司”模式，实行扁平化管理，全面完成“多规合一”改革。新建标准化厂房260万平方米以上，签约率80%以上。坚持差异化、特色化发展，每个县（市）区都要培育2个以上具有特色的优势产业集群。继续推进市经济技术开发区、高新技术园区和北票经济开发区晋升国家级开发区工作。

持续推进项目年建设。推行“项目管家”制度，落实市县领导和部门包扶责任，加快项目落地投产达效。深化投融资体制改革，规范政府投资行为，全力引入社会资本。积极推进北票施可丰生态环保、凌源三沅环保科技、朝阳县光伏平价上网、建平鸿鑫保温材料深加工、喀左信息半导体新材料、双塔新都黄金搬迁改造、龙城加华电子智能飞机零部件等项目。全年实施亿元以上项目230个。

面向全中国、主攻京津冀抓招商。落实县（市）区和园区主体责任，压实部门招商任务，引进一批有实力、有技术、绿色环保和能够“真金白银”投入的企业和项目。以合作共赢、优势互补为切入点，深入开展镇江对口合作和大连对口帮扶活动。发展特色产品，巩固提升龙城、北票等外贸出口基地。积极融入“一带一路”建设，扩大与沿线国家和地区开放合作，稳步推进经贸工作。引进域外资金和实际利用外资均增长12%。

大力发展“飞地经济”。规划建设“飞地经济”专属园区，完善有关政策措施，进一步健全市、县、开发区、乡镇和村多方联动、高效对接机制。探索跨乡镇、跨县域市场化“飞地经济”合作模式，推进产业集群式发展。176个乡镇场街引进“飞地”项目实现全覆盖。

抢抓重大战略机遇。用好用足中央推进东北振兴、省突破辽西北和县域经济发展等政策，积极包装申报项目，推进国家通用航空产业综合示范区、可再生能源综合应用示范区和省级军民融合创新示范区创建工作。向上争取专项资金增长10%。

二、深化改革创新，全力营造良好的投资发展环境

推进供给侧结构性改革。持续巩固去产能成果，稳步推进企业优胜劣汰，加快处置“僵尸企业”。健全完善租购并举的住房制度，合理缩短去化周期，引导房地产市场健康有序发展。完善税收优惠政策和涉企收费清单，扩大直购电交易覆盖范围，推动更大规模减税、更明显降费。

全力优化营商环境。复制推广自贸区改革创新成果，深入推进“放管服”改革，促进权力清单“瘦身”和责任清单“强身”。高质量完成“一网一门一次”改革，实现审批服务“马上办、网上办、就近办”，工程建设项目审批时间压减到90个工作日内。加快商事制度改革，推行证照分离、照后减证。深化“双随机一公开”监管改革，强化事中事后监管。开辟服务企业绿色通道，持续推进专项整治，严肃处理破坏营商环境行为。建立营商环境评价体系，开展营商环境评比活动，营造“人人皆为环境、事事皆为环境”的氛围。

深化国资国企改革。以凌钢集团、朝阳银行、朝柴动力和浪马轮胎等骨干企业为重点，加大对外合资合作力度，实现国有企业混合所有制改革新突破。支持城发、水务、文旅等企业集团发展，推动环境、交投、农垦集团正式运营。推进国有资本授权经营体制改革，组建国有资本投资运营公司。如期完成厂办大集体改革。

加快民营经济发展。强化服务，切实落实支持民营经济发展27条政策措施。实施市场准入负面清单制度，进一步放开民营资本投资领域。组织银企对接，逐步破解小微企业融资难融资贵问题。建立政商交往负面清单，畅通政府与企业联系渠道。依法打击侵犯民营企业合法权益的违法犯罪行为，让民营企业家专心创业、放心投资、安心经营。实施“个转企”“小升规”“规升巨”工程，协同发展大中小微企业。

深化行政管理体制改革。树立过紧日子思想，强化预算绩效管理，调整优化财政支出结构，确保对重点领域和项目支持力度。全面完成事业单位和政府机构改革，深化县乡财政体制改革，扎实推进供销社综合改革。做好向县（市）区放权工作，促进县域和城区经济高质量发展。

大力推进“大学进园区进企业”“双创”基地建设工程。强化科技创新，推动150家以上企业与高校院所合作，转移转化科技成果50项。建立高新技术企业培育库，高新技术企业达到80家。推进“双创”基地建设，吸引域外朝阳人返乡创业和高校毕业生回乡创业。

推进人才建设。狠抓十大引才工程，落实16条人才新政，营造拴心留人的环境。培养造就一支爱家乡、讲奉献的企业家、工匠队伍和劳动者大军，让更多人才在朝阳施展才华、创造财富、实现价值。

三、着力调整产业结构，推动经济高质量发展

积极推进新型工业化。一是加快老企业转型升级，改造提升冶金、汽车及零部件、建材和装备制造等传统产业，确保落地合资合作项目100个以上。二是培育新能源汽车、新材料、节能环保、电子信息、通航等战略性新兴产业，做大石墨烯新材料、航天长峰电源、通美晶体、佛瑞达包装新材料等高新技术企业。支持军民融合产业发展，推进北票市军民融合等项目。大力发展智能制造，推动飞马集团智能改造、美乐电子智能服务等项目加快发展。以质量强市和品牌朝阳建设为推力，不断增强产品竞争力。规模以上工业增加值增长8%。

加快发展现代农业。合理调整“粮经饲”结构，推进优质粮油工程、畜牧业健康养殖工程，以及设施农业、林果业扩量提质增效工程，增加优质绿色农产品供给。全力抓好农产品深加工，实施农产品加工园区提升、现代农业产业园创建和现代农业基地培育工程，推进“一乡一业”“一村一品”，不断提高农业产业化水平。重视培育家庭农场、农民合作社等新型经营主体，大力推进农业共营制发展和标准化建设。开展“互联网、高质量农产品”行动，培育物流和电商企业，包装打造一批高质量农产品和食品品牌。加强动植物疫病防控，确保不发生区域性重大疫情。

提高现代服务业发展水平。坚持生产性服务业和生活性服务业并重，推动服务业提质增效。推进农产品流通体系建设，打通农超、农商对接渠道，新增30家连锁经营主体。加强再生资源、成品油及二手车交易市场管理。发展电子商务、信息、会展、家政服务等新兴产业，引进和培育15家电商企业。狠抓商贸流通品牌建设，做优“老字号”和“辽宁礼物”。丰富节庆消费活动，培育消费载体，打造新消费热点。积极引进外埠金融机构，提高直接融资比重，实现社会融资70亿元。

大力推进文化旅游业发展。深化体制机制创新，加快文化旅游产业市场化进程，培育壮大市场主体。做好文商旅整体规划，加大文旅融合及文创开发力度。推动牛河梁、凤凰山等景区对外合资合作，建好大黑山、热水汤、清风岭、龙源旅游区等文化旅游项目，积极谋划冰雪旅游产业发展。推进全域旅游和乡村旅游，每个县（市）区打造1个乡村旅游示范点、培育1个民宿示范点。加强营销宣传，继续实行“一卡通”年票制度，推进“引客入朝”工程，开展区域旅游合作，打造文化旅游目的地。旅游业总收入增长15%以上。

四、坚持统筹协调，加快推进城乡建设

扎实推进乡村振兴。将产业振兴摆在首要位置，坚持县域重点抓园区、乡镇重点抓“飞地”、村级重点抓集体经济，逐步做大做优县域和乡村经济。创新农村一二三产业融合方式，推进农业与旅游、教育、文化、养生养老等产业深度融合。以美丽乡村、宜居小镇和特色小镇建设为抓手，聚焦垃圾污水处理、村容村貌提升，深入开展农村环境综合整治，创建一批省级生态乡村。进一步深化农村各项改革，抓好农村集体产权制度改革、土地确权颁证收尾和“两区”划定。支持社会力量在农村有序投资创业，带动农民增收。

推进城乡基础设施建设。狠抓中心城区和县城主要道路及景区接驳路提质改造，新建和维修改造“四好农村路”1200公里，确保文化路公铁立交桥交付使用。继续实施环城生态绿廊工程，推进辽西北供水及配套工程和凌源、建平供水工程。抓好朝凌客专、喀赤客专及锦承、叶赤铁路扩能改造，做好锦承铁路叶凌段电化改造工程前期工作。继续推进“厕所革命”，城乡新建和改造公共厕所67座。

加强城市管理。坚持规划引领，依法维护规划严肃性。抓好市容环境综合治理，全力解决停车难停车乱、私搭乱建、老旧小区物业管理不规范、占路经营、卫生死角治理不到位、乱贴广告等群众反映强烈的问题。大力推进违建拆除，进一步巩固沿街烧烤烧纸整治成果。严格落实“三包”责任制。加强智慧城市建设，推动互联网、大数据与城市管理深度融合。

做好进入高铁时代工作。做足“高铁、”文章，打造特色产业带、旅游带和城镇带。坚持点线面结合、分层次推进，持续抓好全域旅游、产业发展、生态环保、城市建设管理、对外宣传推介等短板补齐工作，全力推进交通枢纽等配套设施建设，积极做好相关项目植入。加强地方服务，全力支持高铁可持续安全运营，努力实现共赢发展。

五、加强生态文明建设，切实提高绿色发展水平

强化大气污染防控。严格落实施工扬尘整治措施，完成城区民用散煤清洁替代。加强秸秆禁烧工作，秸秆综合利用率达到86%。继续推进集中供热工程，每个县（市）建设1-2个集中热源。

加强水污染防治。全面落实河长制，开展入河排污口综合整治，实施河道清洁行动。提升工业园区污水处理能力，推进生活污水处理能力扩容改造，抓好加油站双层灌防渗改造，防治地下水污染。打好水源地保护攻坚战，确保水源水质安全。

争创国家森林城市。实施拆违建绿专项整治行动，开展森林“五进”和村屯绿化活动，新增城区绿化面积13万平方米，完成城乡造林绿化60万亩。

保护土壤环境。坚持最严格的耕地保护制度和节约用地制度，切实保护农民合法权益。完成水土流失治理70万亩。推进化肥农药使用零增长行动，实行农用地分类管理，推动土壤环境稳中向好。

强化生态文明制度保障。继续抓好环保督察反馈问题整改，建立健全长效机制。加大领导干部自然资源资产离任（任中）审计、生态环境损害责任追究力度，坚决杜绝破坏生态环境行为。实施非煤矿山综合治理，推进低碳城市试点和气候适应型城市建设试点工作。严把项目准入关，禁止建设高污染、高耗能、低效益项目。

六、树立忧患意识，打好防范化解重大风险攻坚战

积极化解政府债务风险。健全动态监控和风险管控机制，严格控制新增债务，妥善化解存量债务和隐性债务，确保债务风险处于可控区间。

严防养老金支付风险。采取强化征缴管理、加大财政投入力度、积极向上争取等办法筹措资金，严格规范基金支出，确保养老金及时足额发放。

强化金融风险防控。加强对地方法人银行机构、重点债务企业、拖欠银行贷款公职人员、非法集资、民间融资等行业领域规范管理，积极推进农村信用社改制农商行工作，守住不发生系统性、区域性金融风险的底线。

加强和创新社会治理。落实安全生产责任，严控一般事故、遏制较大事故、杜绝重特大事故。做好信访矛盾减存控增工作，健全多元化矛盾纠纷化解机制，减少社会不稳定因素。强化整体联动职能，构建扁平指挥、快速反应的应急管理指挥体系。深入开展扫黑除恶专项斗争，进一步治理行业乱象，不断提升群众满意度。深化治安防控体系建设，提升常态化打击整治力度。狠抓食药品安全监管，继续推进食品安全示范城市创建工作。全面做好第四次全国经济普查和第三次全国国土调查。

七、全力补齐民生短板，切实增强群众获得感幸福感安全感

举全市之力打好脱贫攻坚战。将今年作为决胜年，聚焦“两不愁、三保障”扶贫标准和深度贫困地区基础设施、社会公共服务等短板，以朝阳县摘帽为重点，统筹兼顾非贫困村、贫困边缘村及已脱贫地区脱贫质量巩固提升，狠抓“五个到位”，大力实施“1、10”扶贫举措，确保完成朝阳县摘帽、97个贫困村销号、4万贫困人口脱贫年度目标任务，努力实现高质量脱贫。

加大社会保障力度。把稳就业摆在突出位置，重点解决好高校毕业生、农民工、退役军人等群体就业创业，新增城镇就业1.6万人。加快保障性安居工程建设，继续推进城市棚户区、采煤沉陷区和农村危房改造。完善养老护理体系，努力解决养老难问题。扎实推进社会保险统筹扩面，全力做好城乡低保、社会救助、优抚安置、农民工工资发放、冬季供暖等工作。切实维护老年人、妇女、儿童和残疾人等群体合法权益。

加快发展社会事业。坚持教育优先发展，推进教育信息化建设；新建改扩建城乡公办幼儿园20所，在园儿童普惠率达到75%；提升义务教育均衡发展水平，促进高中教育优质特色发展；加快现代职业教育体系建设，不断深化产教融合；推进朝阳师专转型发展；强化师德师风建设，打造高素质教师队伍。树立大健康理念，深化医药卫生体制改革，加强医联体建设，推进医养结合，增强基本公共卫生服务能力；强化医保管理，遏制小病大治、过度医疗等现象。实施文化惠民工程，完善公共文化服务体系，繁荣发展文化事业和文化产业。统筹发展群众体育、竞技体育和体育产业，创建特色体育名城。落实民族宗教政策，支持民族地区加快发展。做好双拥共建工作。深入推进“七五”普法，提高全社会法治素养。

八、对标人民满意，进一步加强政府自身建设

切实加强政治建设。坚持政府工作讲政治、业务工作重党建，确保党中央国务院、省委省政府和市委决策部署落实落地。认真履行全面从严治党责任，强化“一岗双责”，严格执行中央八项规定精神，严肃查处违纪违法问题，以党建的实际成效保障各项工作顺利推进。

全面推进法治政府建设。自觉接受市人大及其常委会法律监督和工作监督、政协民主监督，主动接受审计监督、社会监督和舆论监督。认真执行市人大及其常委会决议决定，坚持重大事项向市人大及其常委会报告、向市政协通报制度，高质量办好人大代表议案、建议和政协提案。广泛听取各民主党派、工商联、无党派人士和人民团体意见。坚持有诺必践、有诺必行、有诺必果，以政府诚信引领社会诚信。进一步加强政府立法工作。加大政务和信息公开力度，强化8890便民服务平台建设，进一步提高政府公信力。

不断提升执政能力。大胆创新，勇于突破，将“解放思想推动高质量发展”大讨论成果转化为促进发展的动力。正确处理改革发展稳定的关系，高度重视意识形态建设，切实做好群众工作。凝聚干事创业正能量，支持干事者、保护改革者、鼓励担当者。

进一步转变作风。继续开展“重实干、强执行、抓落实”专项行动，切实提升政府执行力。大兴调查研究之风，对表短板弱项，真正解决实际问题。强化督查考核问责，以更实的作风、更严的要求、更高的标准，推动各项工作实现新突破。

各位代表，使命呼唤担当，实干赢得未来。让我们更加紧密地团结在以习近平同志为核心的党中央周围，在省委、省政府和市委的坚强领导下，解放思想、开拓创新，锐意进取、狠抓落实，全面完成今年各项任务，推动朝阳全面振兴和全方位振兴，以优异成绩庆祝中华人民共和国成立70周年！
